# Objectively measured physical activity patterns, sedentary time and parent-reported screen-time across the day in four-year-old Swedish children

**DOI:** 10.1186/s12889-017-4600-5

**Published:** 2017-08-01

**Authors:** Daniel Berglind, Per Tynelius

**Affiliations:** Department of Public Health Sciences, Karolinska Institutet, Tomtebodavägen 18A, 171 77 Stockholm, Sweden

**Keywords:** Accelerometry, Physical activity, Screen-time, Sedentary time, Preschool, Children, Guidelines, Patterns

## Abstract

**Background:**

Physical activity (PA) improves health outcomes accumulating evidence suggests that sedentary time (ST), especially parent-reported screen-time, is associated with negative health outcomes in children. The aim of the present study is to describe levels and patterns of PA and ST across the day and week and activity pattern differences between the sexes, across all weekdays and time spent in and outside the preschool in four-year old children.

**Methods:**

In total 899 four-year old Swedish children who had both complete questionnaire data on screen-time behaviors and objective activity variables and at least 4 days, including one weekend day, with more than 10 h of GT3X+ Actigraph accelerometer wear time data were included in the study. Patterns of PA and ST across the day and week and differences between sexes, weekdays vs. weekend days and time in preschool vs. time spent outside preschool were assessed.

**Results:**

Children engaged in 150 min (SD 73) and 102 min (SD 60) of screen-time on weekend days and weekdays, with 97% and 86% of children exceeding the 1 h guideline for screen-time on weekend days and weekdays, respectively. Accelerometer data showed that boys are more active and less sedentary compared with girls and both sexes were more active and less sedentary on weekdays compared with weekend days, while parent-reported data showed that boys engage in more screen-time compared with girls. Children accumulated 24.8 min (SD. 19) MVPA during preschool time and 26.6 min (SD. 16) outside preschool hours on weekdays, compared with 22.4 min (SD. 18) MVPA during preschool time and 25.3 min (SD. 22) outside preschool hours on weekend days.

**Conclusions:**

Four-year old Swedish children display different activity patterns across the day on weekdays compared to weekend days, with preschool hours during weekdays being the most active segments and preschool hours during weekend days being the least active segments of the day.

**Electronic supplementary material:**

The online version of this article (doi:10.1186/s12889-017-4600-5) contains supplementary material, which is available to authorized users.

## Background

Reducing sedentary time (ST) and physical inactivity are among the most important global health challenges of the twenty-first Century [1]. There is a large body of evidence which suggests that decreasing ST is associated with lower health risk in children [2] and that physical activity (PA) in preschool children improves health outcomes [3]. Children become more sedentary with age [4] and furthermore accumulate their sedentary time in increasingly prolonged bouts [5]. Levels of ST and PA throughout childhood are to a great extent not according to recommendations and both ST and PA track from early childhood into adolescence [6]. This, highlights the importance of establishing recommended levels of PA and ST during early childhood.

Accumulating evidence suggests that the amount of time children spend sedentary may be associated with increased risk of developing metabolic disease independent of moderate to vigorous PA (MVPA) and obesity [7–9], at least when subjective measures such as screen-time are used. On the contrary, observational studies, with objectively measured ST, investigating the effects of prolonged ST and breaks in ST on health outcomes in the pediatric population have failed to detect an association between breaks in ST or sedentary bout length with metabolic disease risk [5, 9–11]. Owing to the evident association between screen-time and several health outcomes [12], government authorities have created specific screen-time guidelines, advocating no more than 1 h of screen-time per day, for children aged two- to four-years of age [13].

Most previous research assessing activity levels in preschool children focus on average activity levels throughout the week. Nevertheless, recent evidence suggests that levels of PA and ST differ noticeably over the course of the day and week [14]. Describing patterns of PA and ST throughout the day provides information when children are less active and thus susceptible to efforts to increase activity. The present study aims to describe average levels and patterns of PA and ST across the day and week and activity pattern differences between time spent in and outside the preschool, using hour specific accelerometer data, in a population-based sample of four-year old Swedish children. In addition, parent-reported screen-time behaviors and accelerometer measured bouts of ST in different lengths will be assessed, which will provide detailed information on how high- and low-intensity activities are distributed across the day and week.

## Methods

### Study design, setting and participants

The present cross-sectional study included four-year-old children from the population-based PRIMROSE trial. The study area, including larger cities, medium sized cities and countryside with low population density, covers approximately 37% of the Swedish population and is fairly representative of Sweden. Recruitment for the PRIMROSE trial started in 2008 and the final follow-up data collection was completed in early 2015. In total, 1369 children were enrolled and 1148 (84%) completed the study. All families signed forms giving their informed consent prior to inclusion in the trial. The PRIMROSE trial was approved by the Stockholm Regional Ethical Review Board (2006/525–31/2) and has been registered as a trial (ISRCTN16991919). A detailed protocol of the PRIMROSE trial, including a consolidated standards of reporting trials diagram, has previously been described [15] and the results from the trial, showing no differences in levels of PA and ST at follow-up, have been published elsewhere [16].

### Anthropometric measurements

The participating children’s height and weight were assessed with validated scales and stadiometers (SECA™) by qualified nurses. Body mass index (BMI) was defined as weight in kilograms divided by height in meters squared. International definitions by Cole et al. were used to classify children’s body sizes as normal weight, overweight or obese [17]. Since BMI was rarely measured at exactly 4 years of age, a non-parametric regression method (kernel smoothing) was used to estimate BMI at 4 years of age [18].

### Physical activity and sedentary time measurements

The Actigraph GT3X+ accelerometer, which has been shown to accurately assess activity levels in young children [19, 20], was used to measure PA and ST. The accelerometer, along with a detailed protocol on how to use it, was sent by mail to the families. Children were instructed to wear the accelerometer on their right hip, attached by a strap, all waking hours for seven consecutive days, and then send it back to the researchers via mail. A sampling rate of 60 Hz sensitivity was used and data was downloaded in 10 s epochs and further aggregated and analyzed in 60 s epochs [21]. We analyzed all three axes (x, y and z) vector magnitude (V_m_) activity counts, calculated as V_m_ = √ (X^2^ + Y^2^ + Z^2^).

Alongside the accelerometer measurements a questionnaire on screen-time and other activities performed throughout the week was sent to the parents. The questionnaire included questions on how many minutes per day, distinct from preschool hours, the children engaged in the following activities: play outdoors, play indoors, playing video games and watching TV, on both weekdays and weekend days. The questions were phrased as: *“How much time does your child spend playing outdoors/indoors, playing video games (question 1) and watching TV (question 2)”* on weekdays and weekend days, respectively. There were eleven answer options: 1 = 0 h/day, 2 = 0.5 h/day, 3 = 1 h/day, 4 = 1.5 h/day, 5 = 2 h/day, 6 = 2.5 h/day, 7 = 3 h/day, 8 = 3.5 h/day, 9 = 4 h/day, 10 = 4.5 h/day 11 = 5 or more hours/day. In addition, the questionnaire included questions on time spent at preschool on a day to day basis, and parental education.

Eight-hundred-ninety-nine children provided valid objectively measured PA [21] defined here as at least 10 h per day for 4 days or more, including both weekdays and at least one weekend day as well as time spent in and outside preschool hours. In this study, 899 children who had both complete questionnaire and accelerometer data were included. Hours between 9 p.m. and 7 a.m. were excluded from the analyses as sleeping hours, based on parent parent-reported questionnaire data, which is in accordance with data showing that four-year old children normally sleep during this time-period [22]. Non-wear time was defined as 60 consecutive minutes with zero counts, allowing for two-minute interruptions with non-zero counts [23]. Furthermore, we calculated ST occurring in 10-min, 20-min and 60-min bouts, since bouts lasting for longer than 10 min have been shown to be associated with negative health outcomes in children [9, 24]. Wear-time and bouts were computed using the *“PhysicalActivity”* and *“Accelerometry”* R-packages, respectively (https://cran.rproject.org).

### Outcome variables

Outcome variables were different levels of PA and ST segmented across the day expressed as average counts per minute (cpm), time spent in different intensities of PA and total and bouts of ST (Table 1). Additional outcome variables included screen-time and activities performed distinct from preschool time, expressed as minutes per day (Table 2). PA and ST were calculated based on intensity cut-offs developed specifically for the GT3X+ accelerometer, using V_m_ activity counts, in four-year-old children [25]. ST was calculated as any minute showing less than 820 cpm. Light PA (LPA) was defined as 820–3907 cpm, MVPA as 3908–6111 cpm and vigorous PA (VPA) as 6112 cpm or more.

**Table 1:** Descriptive characteristics of four-year old children’s physical activity (*N* = 899)

Characteristics	All days Mean (SD)	Weekend day Mean (SD)	Weekday Mean (SD)
Questionnaire PA			
Play outdoors (minutes/day)	136.4 (70.6)	182.6 (85.4)	90.1 (73.1)
Play indoors (minutes/day)	213.6 (84.3)	253.9 (88.7)	173.4 (103.7)
Playing video games (minutes/day)	26.4 (36.9)	33.3 (44.3)	19.5 (32.5)
Watching TV (minutes/day)	99.3 (45.4)	116.7 (52.4)	81.9 (46.4)
Total screen time (minutes/day)	125.7 (61.3)	150 (72.7)	101.5 (59.7)
Accelerometer measured PA			
Wear time (hours/day)	12.2 (0.5)	12.0 (0.8)	12.3 (0.6)
Total PA (cpm/day)	1485.5 (271.6)	1427.2 (340.7)	1507.5 (295.3)
LPA (minutes/day)	361.2 (45.1)	341.2 (58.0)	368.7 (48.9)
MVPA (minutes/day)	50.4 (20.9)	47.7 (25.7)	51.4 (22.6)
VPA (minutes/day)	10.5 (7.9)	10.4 (10.7)	10.5 (8.9)
ST (minutes/day)	321.4 (53.3)	330.8 (71.3)	317.8 (55.9)
10 min ST bout (minutes/day)	286.1 (65.1)	298.5 (88.7)	281.3 (67.9)
20 min ST bout (minutes/day)	165.2 (58.1)	176.5 (81.6)	160.8 (60.9)
60 min ST bout (minutes/day)	51.0 (38.7)	63.5 (67.0)	46.2 (40.1)

**Table 2:** Questionnaire data on screen-time behaviors and activities performed separate from preschool hours on weekdays and during all waking hours on weekend days (*N* = 899). Test for difference between girls and boys from multiple linear regression model (GEE)

Characteristics	Girls Mean (SE)	Boys Mean (SE)	Girls vs. boys Difference (95% CI)^a^
Weekday			
Play outdoors (minutes/day)	85.1 (4.2)	95.9 (4.4)	−10.8 (−22.2, 0.6)
Play indoors (minutes/day)	176.0 (6.1)	171.6 (4.3)	4.4 (−9.1, 18.0)
Playing video games (minutes/day)	16.1 (1.4)	24.5 (1.2)	−8.4 (−11.8, −4.9)
Watching TV (minutes/day)	85.8 (2.4)	78.9 (2.8)	6.9 (−0.5, 14.2)
Total screen time (minutes/day)	100.8 (2.8)	101.9 (3.2)	−1.1 (−9.7, 7.5)
Weekend day			
Play outdoors (minutes/day)	174.7 (4.6)	189.3 (4.8)	−14.6 (−28.6, −0.6)
Play indoors (minutes/day)	256.5 (6.2)	251.9 (3.8)	4.6 (−10.0, 19.2)
Playing video games (minutes/day)	27.1 (1.7)	40.4 (1.8)	−13.3 (−18.4, −8.2)
Watching TV (minutes/day)	118.6 (2.3)	115.1 (2.7)	3.5 (−3.0, 9.9)
Total screen time (minutes/day)	146.0 (3.5)	155.8 (3.5)	−9.9 (−18.4, −1.3)

^a^Adjusted for wear time and parental education, *SE* standard error, *CI* confidence interval

### Exposure variables

Time of day and week as well as time of day were obtained from the accelerometer output. Each day was split into two periods: time spent in pre-school (9 a.m. to 3 p.m.) and time outside pre-school hours (7 a.m. to 9 a.m. and 3 a.m. to 9 p.m.). These time periods were chosen based on the questionnaire data on children’s time spent in preschool.

### Statistical analyses

**Fig. 1:**
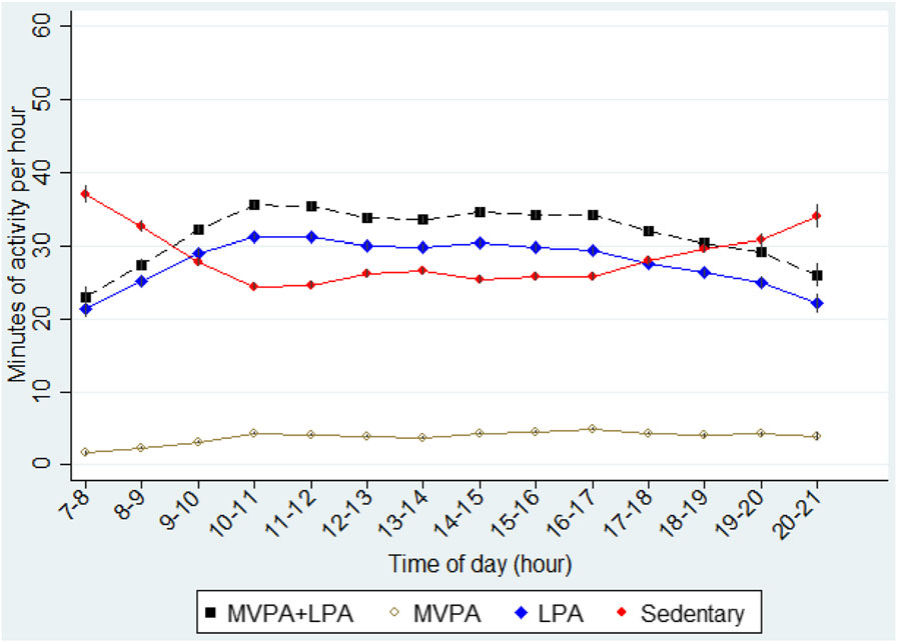
Four-year-old children’s average hourly activity patterns on weekend days (*n* = 899)

**Fig. 2:**
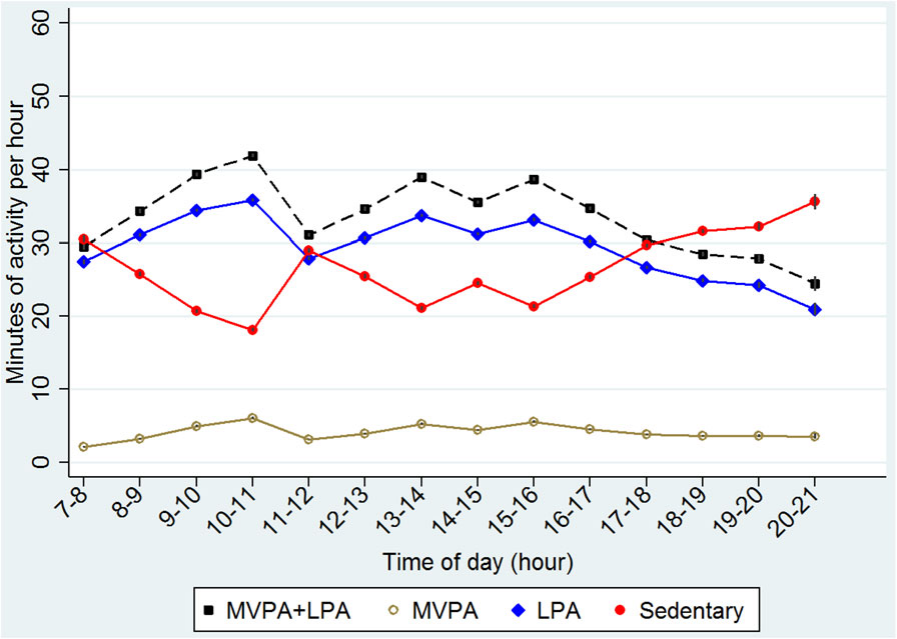
Four-year-old children’s average hourly activity patterns on weekdays (*n* = 899)

Linear regression models using generalized estimating equations (GEE) were used for comparisons between sexes (Tables 2 and 3) as well as for comparisons between periods within children, weekday vs. weekend days and preschool hours vs. outside preschool hours (Tables 4 and 5). All analyses were adjusted for wear-time, and tests between sexes was adjusted for parental education by using GEE with robust variance, which also accounts for slight non-normality. Further adjustment for treatment group did not affect any of the results and was therefore left out. Comparisons between periods within children were carried out using paired t-tests. These differences within children are also controlled for fixed factors by design. Children’s average hourly activity patterns with minutes spent in different intensities of PA and ST on an hour to hour basis during all waking hours were also calculated (Figs. 1 and 2). All analyses were conducted using Stata 14.1 (StataCorp, College Station, TX, USA).

**Table 3:** Accelerometer measured time spent in different intensities of physical activity (PA), sedentary time (ST) and bouts of ST in four-year old children (*N* = 899). Test for difference between girls and boys from multiple linear regression model (GEE)

Time spent in different levels of PA	Girls Mean (SE)	Boys Mean (SE)	Girls vs. boys Difference (95% CI)^a^
Wear time (hours/day)	12.6 (0.03)	12.7 (0.04)	−0.13 (−0.23, −0.03)
Total PA (cpm/day)	1412.0 (15.7)	1497.4 (10.9)	−85.4 (−121.7, −49.2)
LPA (minutes/day)	362.4 (2.6)	372.1 (2.1)	−9.8 (−16.2, −3.4)
MVPA (minutes/day)	45.0 (1.0)	55.9 (0.9)	−10.9 (−13.4, −8.4)
VPA (minutes/day)	10.3 (0.4)	10.7 (0.4)	−0.4 (−1.4, 0.7)
ST (minutes/day)	347.4 (3.5)	334.5 (2.7)	12.9 (3.9, 21.8)
10 min ST bout (minutes/day)	309.1 (4.5)	300.9 (3.1)	8.2 (−2.7, 19.2)
20 min ST bout (minutes/day)	165.3 (4.0)	164.8 (2.7)	0.5 (−8.6, 9.5)
60 min ST bout (minutes/day)	49.3 (2.3)	51.8 (2.3)	−2.5 (−8.3, 3.4)

^a^Adjusted for wear time and parental education, *SE* standard error, *CI* confidence interval

**Table 4:** Accelerometer measured time spent in different intensities of physical activity (PA), sedentary time (ST) and bouts of ST in four-year-old children (*N* = 899). Test for differences within children from paired t-test

Time spent in different levels of PA	Weekend day Mean (SE)	Weekday Mean (SE)	Weekend day vs. weekday Difference (95% CI)*
Wear time (hours/day)	12.0 (0.03)	12.3 (0.02)	−0.30 (−0,36, −0.24)
Total PA (cpm/day)	1427.2 (11.9)	1507.5 (10.3)	−80.3 (−102.4, −58.2)
LPA (minutes/day)	341.2 (2.0)	368.7 (1.7)	−27.4 (−31.2, −23.6)
MVPA (minutes/day)	47.7 (0.9)	51.4 (0.8)	−3.7 (−5.3, −2.1)
VPA (minutes/day)	10.4 (0.4)	10.6 (0.3)	−0.2 (−0.9, 0.6)
ST (minutes/day)	330.8 (2.5)	317.8 (1.9)	13.0 (8.6, 17.4)
10 min ST bout (minutes/day)	298.5 (3.1)	281.4 (2.4)	17.2 (11.8, 22.6)
20 min ST bout (minutes/day)	176.5 (2.8)	160.8 (2.1)	15.6 (10.5, 20.8)
60 min ST bout (minutes/day)	63.5 (2.3)	46.2 (1.4)	17.2 (12.7, 21.8)

*SE* standard error, *CI* confidence interval

**Table 5:** Minutes per hour spent in light physical activity (LPA), moderate-to-vigorous physical activity (MVPA), vigorous physical activity (VPA) and sedentary time (ST) during preschool hours 9 a.m.-3 p.m. and outside preschool hours 7 a.m. -9 a.m. and 3 p.m. -9 p.m. (*N* = 899). Test for differences within children from paired t-test

Time spent in different levels of PA	Outside pre-school hours Mean (SE)	Pre-school hours Mean (SE)	Outside pre-school hours vs. pre-school hours Difference (95% CI)
Weekday			
LPA (minutes/h)	28.6 (0.2)	32.2 (0.2)	−3.7 (−3.9, −3.3)
MVPA (minutes/ h)	4.0 (0.1)	4.6 (0.1)	−0.6 (−0.7, −0.5)
VPA (minutes/ h)	0.9 (0.03)	0.9 (0.03)	0.0 (−0.1, 0.0)
ST (minutes/ h)	27.4 (0.2)	23.2 (0.2)	4.2 (3.9, 4.6)
Weekend day			
LPA (minutes/h)	27.3 (0.2)	30.3 (0.2)	−3.0 (−3.5, −2.6)
MVPA (minutes/ h)	4.1 (0.1)	4.0 (0.1)	0.2 (−0.0, 0.4)
VPA (minutes/ h)	0.9 (0.04)	0.8 (0.03)	0.1 (0.0, 0.2)
ST (minutes/ h)	28.6 (0.2)	25.7 (0.2)	2.9 (2.3, 3.4)

*SE* standard error, *CI* confidence interval

## Results

### Sensitivity analysis

Children excluded due to not providing a valid accelerometer measure or questionnaire data (*n* = 326) had a lower prevalence of parental post-secondary education (*p* < 0.001) and higher prevalence of obesity (*p* = 0.006) compared with those included in the study (Additional file 1: Table S1). We further included children having six or more days and more than 12 h of wear time per day (*n* = 761) in all analyses. However, findings were not affected significantly in any of the analyses presented.

### Main analyses

A total of 899 (93%) children had valid accelerometer data (mean 6.5 (SD 0.7) days), with a mean wear time of 12.2 (SD 0.5) hours per day. Ninety-four percent of the children (956) attended preschool full-time, on average 6.4 h (SD 10.1) per day for 4.7 days (SD 0.9) per week. The current PA guidelines of 180 min of total PA, at any intensity, for children zero to four-years of age, [26] was met by all children on both weekdays and weekend days. On the other hand, intense specific guidelines advocating 60 min or more of daily MVPA was met by 33% and 22% on weekdays and weekend days respectively. Descriptive physical activity characteristics of the study population are presented in Tables 1 and 2. All screen-time and activity variables presented in Table 1 differed significantly (*p* < 0.001) between weekdays and weekend days. On weekdays and weekend days, 86% and 97% of children respectively, engaged in more than the recommended maximum of 1 h of screen-time per day. Children engaged in 150 min (SD 73) of screen-time throughout the day on weekend days and 102 min (SD 60) outside preschool hours on weekdays. Total screen-time did not differ between the sexes, however, boys played more video games on both weekdays (*p* < 0.001) and weekend days (*p* < 0.001), compared with girls.

Average daily time spent in different levels of PA and ST and bouts of ST on weekdays and weekend days are presented in Table 1. Differences between girls and boys using questionnaire data on screen-time behaviors and activities performed separate from preschool and are presented in Table 2, whereas accelerometer measured time spent in different intensities of PA, ST and bouts of ST and differences between sexes and weekdays and weekend days are presented in Tables 3 and 4, respectively. Boys spent more time in MVPA and less time sedentary (not in bouts of ST) compared with girls and both sexes were more active and less sedentary on weekdays compared with weekend days. Children spend 45% of the day in ST on weekdays and 50% on weekend days, whereas boys accumulate 43% and girls 46% of the day as ST during weekdays.

Compared with girls, boys engaged in more MVPA during preschool hours on weekdays (diff = 1.0 (95% CI. 0.7, 1.4) and weekend days (diff = 0.7 (95% CI. 0.4, 1.1) and were less sedentary during preschool hours on weekdays (diff = −1.3 (95% CI. -2.0, −0.6) and weekend days (diff = −1.6 (95% CI. -2.5, −0.7). In addition, compared with girls, boys engaged in more MVPA outside preschool hours on weekdays (diff = 0.8 (95% CI. 0.6, 1.0) and weekend days (diff = 1.1 (95% CI. 0.7, 1.4) and were less sedentary outside preschool hours on weekdays (diff = −1.5 (95% CI. -2.2, −0.7) and weekend days (diff = −1.0 (95% CI. -2.0, −0.08).

Time spent in 10, 20 and 60 min bouts of ST differed significantly between weekdays and weekend days for both sexes (Table 4). On weekdays, children spent 37%, 21% and 9% of the day in ST bouts of 10, 20 and 60 min, respectively. On weekend days 41%, 25% and 10% of the day was spent in ST bouts of 10, 20 and 60 min, respectively.

Children’s average activity for each hour across all waking hours on weekend days and weekdays are presented in Figs. 1 and 2, respectively. Children became increasingly sedentary and spend less time in MVPA after preschool hours (3 p.m.) until sleep. The increase in ST after 3 p.m. was more prominent on weekdays compared to weekend days. On average, children accumulated 24.8 min (SD. 19) MVPA during preschool time and 26.6 min (SD. 16) outside preschool hours on weekdays, compared with 22.4 min (SD. 18) and 25.3 min (SD. 22) MVPA during the same time periods, respectively, on weekend days (Table 5). On weekdays, children accumulated 48% and 45% of daily total MVPA and ST, respectively, during preschool hours. During the same time period on weekend days, children accumulated 47% and 46% of daily total MVPA and ST, respectively. In addition, children spent 7.6% and 6.5% of the time during preschool hours in MVPA on weekdays and weekend days, respectively, and accumulated significantly more time in MVPA (*p* < 0.001) and less ST (*p* < 0.001) during preschool hours on weekdays compared with the same time period on weekend days.

## Discussion

The present study examined average levels and patterns of PA and ST across the day and week and screen-time behaviors in a population-based sample of four-year old Swedish children. Our findings showed that children have different daily patterns of PA and ST on weekdays compared with weekend days. Children were more active and less sedentary during preschool hours on weekdays compared with hours spent outside preschool. Conversely, children were less active and more sedentary from 9 a.m. to 3 p.m. on weekend days compared with 7 a.m. to 9 a.m. and 3 a.m. to 9 p.m. Furthermore, screen-time behavior differed significantly over the week with 86% and 97% of children engaging in more than the recommended maximum of 1 h of screen-time per day on weekdays and weekend days, respectively. The present study provides novel information on how activity and sedentary patterns differ across the day and throughout the week in a population based sample of young Swedish children. These objectively measured time-specific observations may be important for intervention development, targeting periods when children are sedentary and less active. For example, after preschool hours on weekdays and from 9 a.m. to 3 p.m. on weekend days.

Overall, levels of PA and ST observed in the present study are similar to other international studies with objective measured PA and ST. In accordance with findings in the present study, a study on 593 four-year old British children using the Actiheart accelerometer, indicated that children are less active and more sedentary outside preschool hours [14]. However, preschool hours differ substantially between UK (usually 12–5 p.m.) and Sweden (9 am.-3 p.m.) and only 45% of the children in the study by Hesketh et al. attended preschool full-time compared with 94% in the present study. The study by Hesketh et al. used a different accelerometer and only used 1 day of accelerometer data as a valid measure of children’s habitual PA with no analyses of activity patterns across the week, which makes comparability with the present study somewhat limited. A similar study on 703 Australian three- to five-year old preschool children showed that children are highly sedentary and engage in low levels of MVPA in early afternoon on weekdays and around midday on weekends [27]. In contrast to findings in the current study, ST was the lowest and participation in MVPA was the highest in children from afternoon until the evening on weekdays and on weekends. However, the study by van Cauwenberghe et al. used a different accelerometer and children attended preschool fewer hours and to less extent compared to the present study. The increase in MVPA and decrease in ST from awakening until startf of preschool at 9 a.m., found in the present study, may indicate that children routinely use active transportation to the preschool. In addition, the sharp increase in ST from 11 a.m., seen in Fig. 2, may to some extent be explained by the fact that most Swedish preschools serve lunch, which is a sedentary activity, at 11 a.m.

A recent review on 1485 preschooler’s activity levels, comprising 91 preschools, measured with accelerometers or direct observation, showed that children three- to five-years of age spent approximately 3% of the preschool day in MVPA and the majority of the day (>50%) sedentary [28]. This is substantially lower than the 7.6% MVPA, and somewhat higher than the 48% ST, presented in the present study. However, the review included studies from several countries, with somewhat different childcare policies possibly impacting levels of PA, where children on average spend 22 h per week in childcare, which is considerably lower than in the present study.

Objectively measured activity data on Swedish preschool children is sparse. A small Swedish sample of four-year old children showed that children spend 19 out of 445 min (4.3%) in MVPA during preschool hours [29]. However, the study sample was small (*n* = 24) and the authors only analyzed the Vt axis in 15 s epochs from a uni-axial accelerometer, which may oversight preschool children’s sporadic activity patterns [30].

Multiple factors, including measure of PA and ST, data processing and the population studied contribute to the large discrepancies observed across studies. Studies using accelerometers to assess activity in preschool children often use different cut-points to define different intensities of PA and ST. The majority of studies assessing activity with accelerometers in preschool children use uni-axial accelerometers only analyzing the vertical (V_t_) axis [31]. However, a tri-axial accelerometer, such as the GT3X+, may better estimate intensity from free-living daily activities [32]. Several studies assessing ST in preschool children use a cut-point of <100 cpm for the V_t_ axis. Conversely, when analyzing tri-axial accelerometer data from the V_m_ axes, higher cut-points are needed to classify ST [31]. In short, varying data processing protocols and lack of raw cpm comparison of prevalence estimates in young children’s activity levels make comparability between studies, and compliance with guidelines, challenging [33].

The present study observed that children spend a large proportion of the day in LPA, both on weekdays and weekend days as well as during and outside preschool hours. However, health benefits from LPA in preschool children are not fully understood. Studies in four-year old children have shown that MVPA, and not LPA, is positively associated with bone density [34] and lower fat mass [22]. Indeed, the importance of activity intensity requires further investigation to direct future activity guidelines for young children.

Substantial evidence indicates that girls on average are less active and more sedentary compared with boys [4]. This was also observed in the present study. However, this study adds to the current literature by emphasizing that these differences also vary across the day and week. Activity levels between the sexes were most apparent outside preschool, especially during weekdays, indicating that girls are relatively more active and less sedentary than boys in preschool, and vice versa less active and more sedentary when interacting with their parents. Substantial evidence indicates that parent to child correlates of PA is stronger within the same sex, that is father’s levels of PA correlate stronger to boy’s activity levels [35], whereas mother’s levels of PA correlate stronger with girls activity levels [36]. These activity pattern differences across the day between the sexes may direct future interventions to consider a time-based focus to differently target girls and boys. For example, engaging girls in active play during preschool hours and emphasizing maternal PA on both weekdays and weekend days.

Data on screen-time from the present study showed that the majority of children engaged in more than the recommended maximum of 1 h of screen-time per day [37]. This is a higher prevalence compared with a recent population based Canadian study showing that 64% of children aged two- to four-years of age exceeded the maximum screen-time recommendations [38]. However, these differences in prevalence of screen-time may be explained by age differences between the study populations. The high prevalence of screen-time in the present study is alarming, since substantial evidence from a recent systematic review, in children aged zero- to four-years of age, indicates that screen-time, but not objectively measured ST [2], is consistently negatively associated with health outcomes in a dose-response manner [12].

Important to consider is that the present study only measured screen-time outside preschool hours. A recent systematic review on the prevalence of screen-time behaviors in children during preschool hours reported that children on average spend 1 h per day sedentary in front of a screen [39]. Thus, the prevalence of total daily screen-time on weekdays in the present study may be higher than reported. In addition, the present study did not measure other forms of screen time (i.e. mobile phones, tablets etc.) besides TV viewing and computer games. Possibly, the observed screen-time prevalence of 2.5 h per day on weekend days is a more representative prevalence estimate of average screen-time behavior in Swedish four-year old children.

### Strengths and limitations

The strengths of the present study a largest study population of Swedish four-year old children entailing objectively measured activity patterns across the day. The study area, including larger cities, medium sized cities and countryside with low population density, covers approximately 37% of the Swedish population and is fairly representative of Sweden with children from all socio-economic strata [15]. However, results from the present study are only generalizable for the Swedish population. In addition, we used accelerometers to assess PA and ST patterns across the day and both measurements and data processing followed best practices [40]. We have used cut-points validated in four-year old children developed specifically for the accelerometer (GT3X+) used in the present study [25]. However, analysis of the Vm axes and higher cut-points to define ST compared with several other studies analyzing the Vt axis limits the comparability with previous research [31]. Additional limitations with using accelerometers to define ST is their inability to separate sitting from standing [41]. Hence, we may have misclassified standing time as ST, which according to the latest definition is not defined as time spent sedentary [42].

We included all children with at least 4 days of valid PA data, including one weekend day, and sensitivity analyses on children with six or more days of PA data did not alter the results. A limitation possibly affecting results presented in the statistical models is the lack of appropriate data at preschool level. Thus, we were unable to adjust your analyses for possible clustering of participants within preschools.

The use of hour-specific data, dividing the day into two time-periods, enables a more detailed description of time-based patterns of children’s activity levels across the day and week. These segments reflect preschool children’s daily differences in activity intensity, both between time spent in and outside the preschool and between weekdays and weekend days. However, time-matched data on children not attending preschool was not available and we were therefore unable to assess what influence preschool attendance had on children’s activity patterns across the day. Nevertheless, this unique hour by hour activity data across the day on both weekdays and weekend days’ pinpoints specific periods throughout the day and week where public health interventions may be more likely to show a beneficial impact on children’s activity levels.

## Conclusion

Four-year old Swedish children display different activity patterns across the day on weekdays compared to weekend days, with preschool hours during weekdays being the most active segments and the same time period during weekend days being the least active segments of the day. In addition, children’s patterns of PA and ST across the day as well as screen-time behaviors differ between weekdays and weekend days. These temporal differences in PA and ST across the day should be taken into account when designing interventions to increase preschoolers’ PA and reduce ST and screen-time behaviors, for example focusing on reducing weekend screen-time behavior and increasing PA between 9 a.m. and 3 p.m. on weekend days and after 3 p.m. on weekdays.

## Additional file

Additional file 1: Table S1. Descriptive characteristics of four-year old children with invalid accelerometer data. (DOCX 46 kb)
