# Supplementary material for: Objectively measured physical activity patterns, sedentary time and parent-reported screen-time across the day in four-year-old Swedish children
Source: BMC Public Health. 2017 Aug 1;18:69. doi: 10.1186/s12889-017-4600-5 (PMC5540346; doi:10.1186/s12889-017-4600-5)
Supplement: Additional file 1: Table S1. — Descriptive characteristics of four-year old children with invalid accelerometer data. (DOCX 46 kb) [file 12889_2017_4600_MOESM1_ESM.docx]

**Supplementary material**

**Table 1.**

Descriptive characteristics of four-year old children with invalid accelerometer data.

| **Characteristics** | **Total sample (n=66) (SD)** | **Girls (n=24) (SD)** | **Boys (n=42) (SD)** |
| --- | --- | --- | --- |
| **Age (years)** | 4.1 (0.08) | 4.1 (0.06) | 4.1 (0.1) |
| **BMI (kg/m^2^)** | 15.9 (1.4) | 16.4 (1.7) | 15.7 (1.0) |
| **Waist circumference (cm)** | 52.7 (3.7) | 53.7 (4.9) | 52.1 (2.6) |
| **Overweight (%)** | 12.5 | 14.8 | 10.1 |
| **Obese (%)** | 3.3 | 4.2 | 2.3 |
